# Antibacterial Effect of Cannabinoids on Bacteria Associated with Persistent Endodontic Infections

**DOI:** 10.3390/ijms262411936

**Published:** 2025-12-11

**Authors:** Cassandra Wieczerza, Haoyan Zhai, Mazin Askar, Zheng Zhou, Susan Paurazas

**Affiliations:** 1 Endodontic Program, Division of Graduate Education, School of Dentistry, University of Detroit Mercy, Detroit, MI 48208, USA; 2 Periodontic Program, Division of Graduate Education, School of Dentistry, University of Detroit Mercy, Detroit, MI 48208, USA; zhyan299@gmail.com (H.Z.); zhouzh1@udmercy.edu (Z.Z.)

**Keywords:** cannabinoids, CBD, CBN, THC, persistent endodontic infection, *Enterococcus faecalis*, *Streptococcus mutans*, *Fusobacterium nucleatum*

## Abstract

Cannabinoids have been shown to have effective antibacterial applications. With the limitations of current intracanal endodontic medicaments and the rise of bacterial resistance, it is important to investigate novel treatment strategies for endodontic infections. The aim of this study was to test the antibacterial efficacy of cannabinoids on bacteria in persistent endodontic infections: *Enterococcus faecalis*, *Streptococcus mutans*, and *Fusobacterium nucleatum*. Planktonic bacteria were exposed to a negative control (no exposure), a positive control (3% NaOCl), and the experimental groups Cannabidiol (CBD), Cannabinol (CBN), and Tetrahydrocannabinol (THC). The Minimum Inhibitory Concentration (MIC) and Minimum Bactericidal Concentration (MBC) were also investigated. Biofilms were cultured and treated with cannabinoids. A crystal violet assay (CVA) and live/dead analysis assessed the biofilm degradation and inhibition, respectively. A statistical analysis was performed using an ANOVA. CBD, CBN, and THC reached a MIC for both *E. faecalis* and *S. mutans* in planktonic forms. The MBC was found for the tested cannabinoids on planktonic *E. faecalis*. No MBC was found for *S. mutans*. The live/dead analysis of *E. faecalis* and *S. mutans* biofilms showed a decrease in the viability of the biofilm with an increased cannabinoid concentration. The CVA revealed that cannabinoids only degrade the *E. faecalis* biofilm. Planktonic *F. nucleatum* had no MIC for tested cannabinoids. Cannabinoids have inhibitory effects on *E. faecalis* and *S. mutans* in the planktonic and biofilm states. No inhibitory effects of *F. nucleatum* were found at tested concentrations of all three cannabinoids. The findings suggest that cannabinoids have distinct antibacterial effects on certain pathogens associated with persistent endodontic infections.

## 1. Introduction

The primary objective of endodontic therapy is to treat pulpal and/or periapical pathosis by eliminating causative microorganisms. This is achieved through chemo-mechanical processes and followed by accurate three-dimensional obturation. This will seal the canal from external sources of microleakage. Eradicating bacteria can present difficulties, as the microbial makeup of each individual is unique and ranges from 100 to 200 of the possible bacterial species in an infected canal [1]. This presents difficulties as the microbial makeup of individuals is unique. Further adding to the bacterial complexity, endodontic infections can be characterized as primary, persistent, or secondary [2]. Over 500 microorganisms have been discovered in pulpal infections; however, a core microbiome of 20–30 organisms has been identified. Secondary infections are less diverse than primary infections and are composed largely of the following phyla: *Enterococcus faecalis* (*E. faecalis*), *Streptococcus* species, *Actinomyces* species, and *Dilaster* species [3]. *E. faecalis* is unique in being the most consistently found organism in persistent infections [4,5,6,7]. A recent scoping review on the microbial profile of persistent endodontic infections identified the most prevalent species as *Enterococcus faecalis*, *Parvimonas micra*, *Porphyromonas endodontalis*, *Porphyromonas gingivalis*, *Prevotella intermedia*, *Dialister invisus*, *Propionibacterium acnes*, *Tannerella forsythia*, and *Treponema denticola* [8]. Even with current root canal disinfection protocols, bacteria persist. In a histological evaluation of teeth with apical periodontitis, biofilms were present in 74% of treated canals [9]. Persistent bacteria may lead to flare-ups following treatment. These microorganisms include *Porphyromonas gingivalis*, *Porphyromonas endodontalis*, *Fusobacterium nucleatum* (*F. nucleatum*), and *Prevotella.* Measures to decrease the flare-up potential include mechanical instrumentation techniques, aseptic conditions, and the use of intracanal medicaments [10]. Calcium hydroxide, however, has been found to have a limited effect on *E. Faecalis* [11,12,13]. The use of antibiotics systemically or within an intracanal medicament has led to an increase in antibiotic resistance. Multidrug-resistant *E. Faecalis* is associated with persistent infections, while beta-lactamase-resistant Gram-negative bacteria have been directly linked to the treatment failure of purulent endodontic infections [14]. Calcium hydroxide, a common intracanal medicament, has been shown to have limitations in effectiveness against *F. nucleatum* persisting after treatment [15]. Since *F. nucleatum* has been shown to persist after treatment with calcium hydroxide, natural additions such as propolis have been tested in combination with calcium hydroxide for increased antibacterial efficacy [16]. The antibacterial efficacy of Cannabinoids, another group of naturally occurring substances has been researched. Cannabinoids have an antimicrobial effect against Gram-positive and Gram-negative bacteria [17,18,19]. Cannabinoids used in medical research include CBD, CBN, and THC. CBD has an inhibitory effect on the release of membrane vesicles from Gram-negative bacteria that impair bacterial cell communication, thus decreasing the antibacterial resistance of biofilms. Cannabinoids added to mouthwash have been proven to have the same antibacterial efficacy as 0.2% chlorohexidine, lending to its antibacterial potential [20]. In addition to antibacterial benefits, cannabinoids have a positive biocompatible effect on the dentin, pulp, and bone regeneration as well as on dental pulp cells [21,22]. With limitations of available intracanal medicaments and the rise of antibacterial resistance, the exploration of novel antimicrobial medicaments is key for future treatment options. The aim of this study was to test the antibacterial efficacy of cannabinoids, CBD, CBN and THC, on bacteria found in persistent endodontic infections: *E. faecalis*, *Streptococcus mutans* (*S. mutans*), and *F. nucleatum*.

## 2. Results

### 2.1. Minimum Inhibitory Concentration (MIC) of CBD, CBN, and THC Against E. faecalis, S. mutans, and F. nucleatum

**Figure 1:**
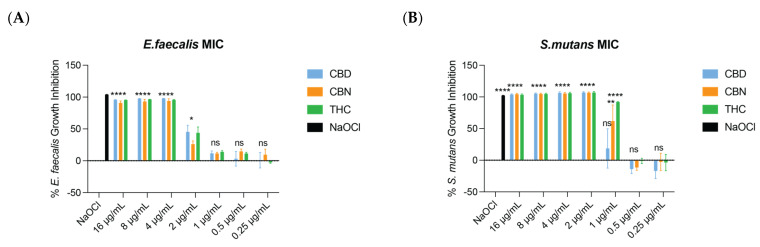
The MIC (90% growth inhibition) of CBD, CBN, and THC for *E. faecalis* and *S. mutans*. (**A**) For *E. faecalis* the MIC of CBD was 4 μg/mL. The MIC of CBN was 4 μg/mL. The MIC of THC was 4 μg/mL. (**B**) For *S. mutans* the MIC of CBD was 2 μg/mL. The MIC of CBN was 2 μg/mL. The MIC of THC was 1 μg/mL. One-way ANOVA followed by Dunnett’s post hoc test. **** *p* < 0.0001; ** *p*< 0.01; and * *p* < 0.05. ns, not significant.

The MIC of CBD, CBN, and THC was determined to be the lowest concentration of each drug that reached 90% growth inhibition. For *E. faecalis*, the MIC for all three cannabinoids was 4 µg/mL (Figure 1A). A one-way ANOVA revealed a significant overall group effect (*p* < 0.0001). The post hoc Dunnett’s test (**** *p* < 0.0001; *** *p* < 0.001; ** *p* < 0.01, and * *p* < 0.05) showed that concentrations ≥ 2 μg/mL significantly differed from the methanol control (*** *p* < 0.001), whereas concentrations ≤ 1 μg/mL showed no significant difference from the methanol control (*p* > 0.05).

**Figure 2:**
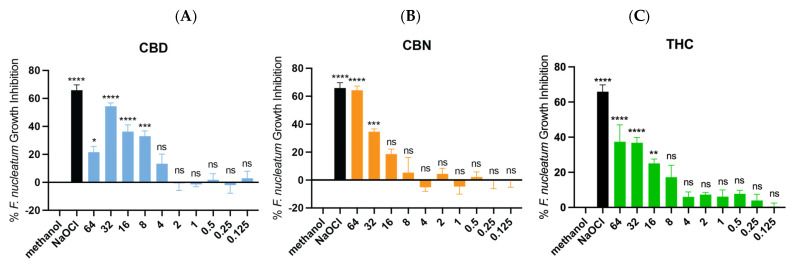
MIC (90% growth inhibition) of CBD (blue), CBN (orange), and THC (green) for *F. nucleatum*. (**A**) No MIC was found for CBD. (**B**) No MIC was found for CBN. (**C**) No MIC was found for THC. One-way ANOVA followed by Dunnett’s post hoc test. **** *p* < 0.0001; *** *p* < 0.001; ** *p*< 0.01; and * *p* < 0.05. ns, not significant.

For *S. mutans*, the MIC of CBD and CBN was 2 µg/mL, and for THC it was 1 µg/mL (Figure 1B). The ANOVA test indicated a statistical difference in inhibition between drug concentrations (*p* < 0.05). For the CBN at 1 µg/mL, the *p* value was <0.01 (**), whereas for THC at 1 µg/mL, the *p* value was <0.0001 (****), indicating a stronger and more statistically robust inhibitory effect of THC at this concentration. No MIC for *F. nucleatum* was reached for the CBD (Figure 2A), CBN (Figure 2B), or THC (Figure 2C) at tested drug concentrations (0.015 µg/mL–64 µg/mL). The one-way ANOVA and post hoc Dunnett’s test (**** *p* < 0.0001; *** *p* < 0.001; ** *p* < 0.01, and * *p* < 0.05) showed that concentrations of CBD, CBN and THC significantly differed from the methanol control for the growth inhibition of *F. nucleatum*, yet no MIC was reached.

### 2.2. Minimum Bactericidal Concentration (MBC) of CBD, CBN, and THC Against E. faecalis and S. mutans

The MBC of CBD, CBN, and THC was determined when no growth was seen upon plating suspensions. For *E. faecalis*, the MBC was 16 µg/mL for CBD and CBN and 64 µg/mL for THC (*p* < 0.05). *S. mutans* suspensions treated with 2 µg/mL through 64 µg/mL of CBD and CBN were plated. Suspensions treated with 1 µg/mL through 64 µg/mL of THC were plated. No MBC was found for tested drugs at all concentrations (*p* < 0.05). The MBC was not studied for *F. nucleatum* since no MIC was found.

### 2.3. Biofilm Degradation

**Figure 3:**
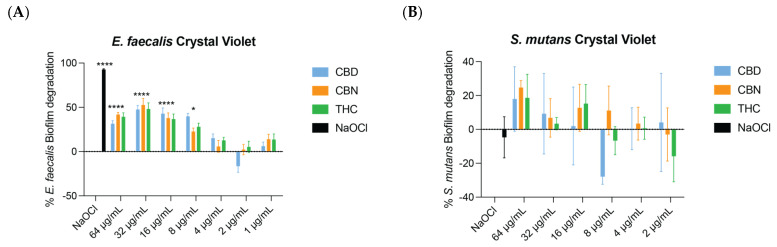
Result of crystal violet assay analyzing biofilm degradation by CBD, CBN, and THC. (**A**) For *E. faecalis*, at a concentration of 32 μg/mL CBD and CBN were able to degrade > 50% of the biofilm. At a concentration of 32 μg/mL THC degraded 48% of the biofilm. (**B**) For *S. mutans*, the crystal violet assay revealed that the biofilm was not degraded to a level of 50% by any cannabinoid at tested concentrations. One-way ANOVA followed by Dunnett’s post hoc test. **** *p* < 0.0001; and * *p* < 0.05.

The crystal violet assay was performed on single-organism biofilms of *E. faecalis* and *S. mutans* to assess the degradation ability of CBD, CBN, and THC. TECAN readings revealed that at 32 µg/mL CBD and CBN degraded >50% of the *E. faecalis* biofilm (*p* < 0.05). While not statistically significant, 32 µg/mL of THC degraded 48% of the *E. faecalis* biofilm. Although a statistical significance was observed at lower concentrations (e.g., 16 μg/mL and 8 μg/mL), the threshold of >50% degradation was only achieved at 32 μg/mL. The asterisks in Figure 3A denote statistical differences compared with the control, while the statement “>50% degradation at 32 μg/mL” refers specifically to the biological effectiveness threshold rather than the statistical significance (Figure 3A). With the *S. mutans* biofilm, no drug was able to degrade it to a level of 50% at tested concentrations (*p* > 0.05) (Figure 3B). A biofilm analysis was not performed on *F. nucleatum* as no MIC was found in the planktonic form.

### 2.4. Biofilm Viability

**Figure 4:**
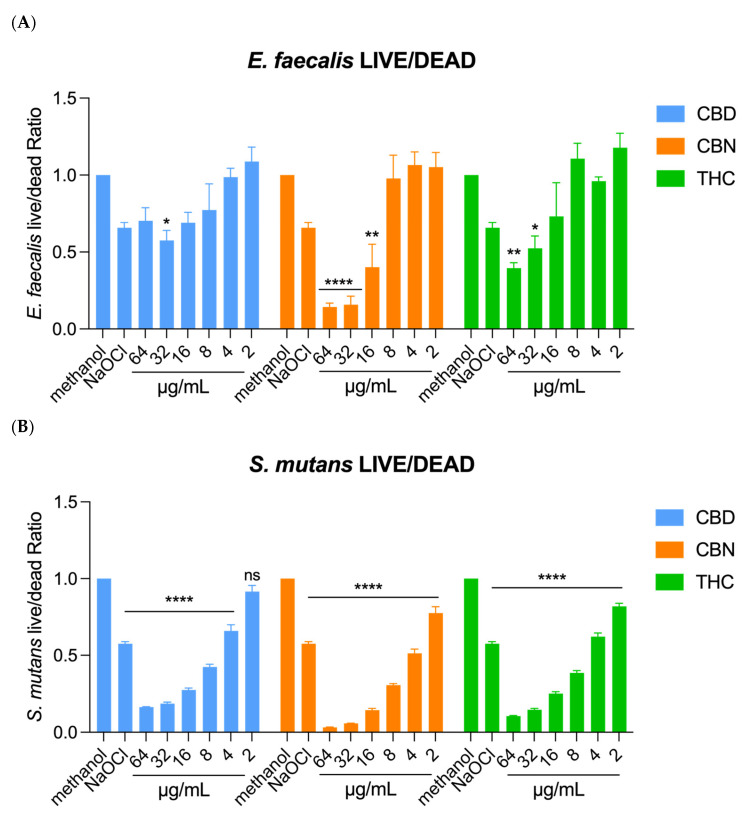
Result of live/dead analysis for evaluating effect of CBD, CBN, and THC on biofilm viability. (**A**) For *E. faecalis*, an increase in all cannabinoid concentrations caused a decrease in biofilm viability. (**B**) For *S. mutans*, an increase in all cannabinoid concentrations caused a decrease in biofilm viability. One-way ANOVA followed by Dunnett’s post hoc test. **** *p* < 0.0001; ** *p*< 0.01; and * *p* < 0.05. ns, not significant.

In regard to the effect of CBD, CBN, and THC on the viability of single-organism biofilms, the live/dead analysis found that for both *E. faecalis* and *S. mutans* as the concentration of all cannabinoids increased, the viability decreased (*p* < 0.05). In both *E. faecalis* and *S. mutans* biofilms, CBN demonstrated the strongest inhibitory activity. CBN produced significant reductions in viability already at 16 μg/mL (**) and reached a very strong significance (****, *p* < 0.0001) at both 32 and 64 μg/mL, indicating a robust and dose-dependent effect. THC showed moderate efficacy, with statistical significance detected at 32 μg/mL (*), which further increased to a higher significance at 64 μg/mL (**), supporting a concentration-dependent increase in the inhibitory effect. In contrast, CBD displayed the weakest response within the tested concentration range, showing only a single significance marker (*) at 32 μg/mL. These results together imply that CBN has the highest antimicrobial potency against both organisms, followed by THC, with CBD exhibiting the lowest overall impact at comparable concentrations (Figure 4A,B).

## 3. Discussion

While a root canal treatment is highly successful, a systematic review on endodontic treatment outcomes using strict criteria found a weighted pooled success rate between 68% and 85% [23]. Persistent bacteria are cited as one of the most common causes of treatment failure [24]. Due to the complexity of the canal system, biofilms may escape chemo-mechanical debridement [25]. Studies have shown that calcium hydroxide, used as an intracanal medicament, has an antimicrobial effect on many endodontic pathogens; however, it is limited against *E. faecalis* [26]. Limitations of the efficacy of calcium hydroxide against *F. nucleatum* have been noted, and alternative intracanal medicaments have been tested. A calcium silicate intracanal medicament has been shown to have superior biofilm efficacy as compared to calcium hydroxide after a 2-week application [27]. In the present research, cannabinoids had an MIC against *E. faecalis* and *S. mutans*. Contrarily, no MIC was reached against *F. nucleatum*. These findings coincide with the published research, which has shown that CBD has a remarkably consistent MIC of 1–4 µg/mL against over 20 g-positive strains. The efficacy against Gram-negative bacteria is limited. Studies have found that by removing the lipid-A chain of LPS in Gram-negative bacteria, CBD can penetrate and inhibit the bacteria [19]. Thus, it can be inferred that the lipid-A chain interferes with CBD’s antibacterial effect. Intracanal antibiotics are used during apexification and regenerative endodontic procedures [28,29,30]. Intracanal antibiotics, most commonly triple antibiotic paste (TAP), have been recommended by the American Association of Endodontists as one of the medicaments to be used during regenerative endodontics [31]. Studies have shown the TAP’s efficacy for the reduction in root canal microorganisms, providing a more appropriate environment for these procedures [32,33]. While valuable, clinicians must be aware of the risks of antibiotic resistance, allergic reaction, damage to vital stem cells, and possible tooth discoloration [34,35,36]. Research has shown that CBD has a very low innate resistance frequency value [19]. In addition to the direct effects of cannabinoids on bacteria, CBD’s efficacy increases when added to colistin, rifampicin, erythromycin, kanamycin, and vancomycin [37]. A CBD addition to bacitracin produced a 64-fold decrease in the MIC, thus increasing its efficacy for Gram-positive bacteria. It was specifically found to affect the bacterial cell membrane and reduce a major cell division gene [38]. While the present study has shown promising results by treating *E. faecalis* and *S. mutans* with cannabinoids, there were limitations to this work. As this was an in vitro study, findings may not reflect the true effects of in vivo conditions. Bacteria were cultured and grown in plates and wells that do not represent the intricate root canal system of a tooth. This study was conducted on individual bacteria and not complex biofilms that are seen in actual endodontic infections. Single-organism biofilms do not accurately represent root canals with persistent infections. The biofilm morphology, bacterial diversity and local environmental factors influence the nature and character of endodontic infections [3]. Additional limitations of in vitro studies include the fact that the in vitro model does not reproduce the complexity of the host response to bacterial biofilms. Drug concentrations can vary in relation to pharmacokinetics, such as absorption and distribution. Future studies could be performed using cannabinoids in an intracanal medicament or possible chemical preparation of the canal. Potential applications include a topical agent for vital pulp therapy or incorporation in an intracanal medicament in cases of pulpal necrosis or persistent endodontic infections. Considerations of the potential risks of local and systemic effects of intraoral applications would need to be evaluated. Oral effects of cannabis use include xerostomia and periodontal disease [39]; however, targeted applications may provide a therapeutic benefit and minimize potential adverse effects A study evaluating the cytotoxic effect of acute and chronic exposure to CBD on gingival fibroblasts and oral keratinocytes found that a concentration > 50 µM was cytotoxic [40]. This correlates to approximately 16 µg/mL, a concentration well above the MIC of 4 µg/mL for *S. mutans* and *E. faecalis*, as determined in our study. In vivo studies would also prove important to determine the efficacy of cannabinoids in an infected root canal system. In vivo studies assessing efficacy with a complex anatomical morphology would also be beneficial. The results of this study represent a promising start to further research identifying the targeted applications of cannabinoids in the effective treatment of persistent endodontic bacterial infections.

## 4. Materials and Methods

### 4.1. Media and Growth Conditions

Standard bacterial pathogens, *Streptococcus mutans* (ATCC 25175, Manasses, VA, USA), *Enterococcus Faecalis* (ATCC 29212), and *Fusobacterium nucleatum* (ATCC 25586), were used, which were obtained from ATCC. *S. mutans* were grown on brain heart infusion (BHI) agar (BD Laboratories, Sparks, MD, USA) and medium in 5% CO_2_ at 37 °C. *E. faecalis* and *F. nucleatum* were grown on brucella broth agar (BD Laboratories, Sparks, MD, USA) with vitamin K (Sigma-Aldrich, St. Louis, MO, USA), hemin (Acros Organics/Fisher Scientific, Waltham, MA, USA) and sheep blood (Hemostat Laboratories, Dixon, CA, USA), (BBA++) agar and brucella broth with vitamin K and hemin (BB++) medium in an anaerobic environment at 37 °C. Anaerobic bacteria growth media were pre-reduced in the anaerobic chamber for 24–36 h prior to inoculation with bacteria.

### 4.2. Growth Curve and Optical Density–Colony-Forming Units

Growth curve and optical density–colony-forming units (OD-CFUs) were examined using published methods [41,42].

### 4.3. Minimum Inhibitory Concentration (MIC) of CBD, CBN, and THC

The MIC of CBD, CBN, and THC (Millipore sigma, Merck KGaA, Darmstadt, Germany) for each bacterial strain was tested in 96-well plates. Drugs were prepared to the following concentrations: 64 μg/mL, 32 μg/mL, 16 μg/mL, 8 μg/mL, 4 μg/mL, 2 μg/mL, 1 μg/mL, 0.5 μg/mL, 0.25 μg/mL, 0.125 μg/mL, 0.06 µg/mL, and 0.03 μg/mL. Each well contained 100 μL of drug solution and 100 μL of bacterial suspension. The final concentrations of *E. faecalis*, *S. mutans*, and *F. nucleatum* were 1.5 × 10^6^ CFU/mL, 0.5 × 10^6^ CFU/mL, and 5 × 10^6^ CFU/mL, respectively. The positive control wells comprised 100 μL 6% NaOCl with 100 μL of bacterial suspension. The negative control wells comprised 100 μL BB++ broth for *E. faecalis* and *F. nucleatum* and 100 μL BHI broth for *S. mutans*. The vehicle control was methanol at the same previously stated concentrations of drugs and 100 μL bacterial suspension. *S. mutans* were placed in incubator at 5% CO_2_ at 37 °C, and *E. faecalis* and *F. nucleatum* were placed in anaerobic hood at 37 °C. The plates were read in the TECAN plate reader. MIC was determined when at least 90% growth inhibition was reached.

### 4.4. Minimum Bactericidal Concentration (MBC) of CBD, CBN, and THC

For MBC determination, suspension from wells at 1×, 2×, 4×, and 8× MIC were plated onto appropriate agar and incubated in appropriate conditions until visible colonies were seen to evaluate bacterial survival. Colonies were then counted. The lowest drug concentration with no colonies was determined to be the MBC.

### 4.5. Biofilm Degradation Assay

Biofilm degradation assays were performed on *E. faecalis* and *S. mutans*. For *E. faecalis*, overnight culture was washed and resuspended in BB++ broth to achieve an optical density (OD) = 0.056. Then 200 μL of resuspended bacteria was added to a 96-well plate and incubated anaerobically at 37 °C for 24 h to form the biofilm. For *S. mutans*, overnight culture was washed and resuspended to make an OD = 0.1. In each well, 50 μL of *S. mutans* suspension, 2 μL of 50% sucrose, and 148 μL of BHI broth was added to 96-well plate and incubated in 5% CO_2_ at 37 °C for 24 h to form the biofilm. After biofilm formation, the supernatant was removed. Drug concentrations of 1, 2, 4, 8, 16, 32, and 64 μg/mL were created, and 200 μL of the drug was added to each well. The positive control was 3% NaOCl, the negative control was appropriate broth, and the vehicle control was methanol at concentrations corresponding to drugs. The plates were incubated for 24 h. Biofilm formation of *E. faecalis* and *S. mutans* was examined using CVA. The treated plates were obtained, and the supernatant was removed. The plate was heat-fixed, and 0.1% crystal violet solution was added to each well. After 15 min, wells were rinsed with water and air-dried. The crystal violet in the biofilm was solubilized with 33.3% acetic acid, and absorbance was measured with TECAN plate reader. Biofilms were considered degradable when a threshold of 50% degradation was reached.

### 4.6. Biofilm Activity Assay

Biofilms of *E. faecalis* and *S. mutans* were pre-formed in 24-well plates under the specified culture conditions and concentration ratios described in Section 4.5. After biofilm formation, the supernatant was removed. Drug concentrations of 1, 2, 4, 8, 16, 32, and 64 μg/mL were created, and 1 mL of the drug was added to each well. The positive control was 3% NaOCl, the negative control was appropriate broth, and the vehicle control was methanol at concentrations corresponding to drugs. The plates were incubated for 24 h. The supernatant was removed, and wells were washed with PBS. Then, 250 μL of Propidium Iodide (PI) and SYTO 9 staining buffer was added to each well. Plates were kept in a dark environment for 15 min then read using the TECAN plate reader to analyze biofilm viability.

### 4.7. Statistical Analysis

All the experimental results were normalized according to controls and analyzed by ANOVA. Data were presented as mean ± SEM. *p* < 0.05 was considered statistically significant. Data were analyzed using a one-way ANOVA followed by Dunnett’s multiple comparisons test to compare each treatment group with the methanol control. Biofilm viability was analyzed with one-way ANOVA with a linear trend analysis (*p* < 0.05).

## 5. Conclusions

Cannabinoids, CBD, CBN, and THC, were found to have an inhibitory effect on two of the three tested bacteria. An MIC as well as an MBC were found for *E. faecalis* in the planktonic state. While no MBC was found for *S. mutans*, an MIC was reached for the planktonic state. With an increase in the cannabinoid concentration there was a decrease in the biofilm viability of *E. faecalis* and *S. mutans*. *E. faecalis* biofilms were shown to be degraded by the cannabinoid treatment. Contrary to these results, cannabinoids had no antibacterial effect on *F. nucleatum*. These findings could influence future studies and applications in the treatment of persistent endodontic infections.

## Data Availability

The original contributions presented in this study are included in the article. Further inquiries can be directed to the corresponding author.
